# No Effects of Photobiomodulation on Prefrontal Cortex and Hippocampal Cytochrome C Oxidase Activity and Expression of c-Fos Protein of Young Male and Female Rats

**DOI:** 10.3389/fnins.2022.897225

**Published:** 2022-05-06

**Authors:** Alba Gutiérrez-Menéndez, Juan A. Martínez, Marta Méndez, Jorge L. Arias

**Affiliations:** ^1^Laboratory of Neuroscience, Department of Psychology, University of Oviedo, Oviedo, Spain; ^2^Instituto de Neurociencias del Principado de Asturias (INEUROPA), Oviedo, Spain; ^3^Instituto de Investigación Sanitaria del Principado de Asturias (ISPA), Oviedo, Spain; ^4^Electronic Technology Area, University of Oviedo, Gijón, Spain

**Keywords:** photobiomodulation, development, brain stimulation, nervous system, low-level light therapy

## Abstract

The role of light in our biological processes and systems is extensively known. In addition, the use of light devices has been introduced in the field of healthcare as an opportunity to administer power light at specific wavelengths to improve our body functions and counteract light deficiency. One of these techniques is photobiomodulation (PBM), which uses red to infrared light in a non-invasive way to stimulate, heal, regenerate, and protect tissue. The main proposed mechanism of action is the stimulation of the cytochrome c oxidase (CCO), the terminal enzyme in the mitochondrial electron transport chain. PBM has achieved positive effects on brain activity and behavioral function of several adult animal models of health and disease, the potential use of this technique in developing stages is not surprising. This research aims to examine the effects of PBM on the prefrontal cortex and hippocampus of 23 day-old healthy male (*n* = 31) and female (*n* = 30) Wistar rats. Three groups of each sex were used: a PBM group which received 5 days of PBM, a device group submitted to the same conditions but without light radiation, and a control basal group. CCO histochemistry and c-Fos immunostaining were used to analyze brain metabolic activity and immediate early genes activation, respectively. Results displayed no metabolic differences between the three groups in both sexes. The same results were found in the analysis of c-Fos positive cells, reporting no differences between groups. This research, in contrast to the PBM consequences reported in healthy adult subjects, showed a lack of PBM effects in the brain markers we examined in young healthy rat brains. At this stage, brain function, specifically brain mitochondrial function, is not disturbed so it could be that the action of PBM in the mitochondria may not be detectable using the analysis of CCO activity and c-Fos protein expression. Further studies are needed to examine in depth the effects of PBM in brain development, cognitive functions and postnatal disorders, along with the exploration of the optimal light parameters.

## Introduction

The role of light in our biological processes and systems is extensively known. It is a potent regulator of body functions that can activate other complex biological pathways. Light controls our sleep-wake cycles, our circadian rhythms, impacts our mental health, and provides us with essential vitamins (Dompe et al., 2020; Bertani et al., 2021). In this regard, the use of light devices has been introduced in the field of healthcare as an opportunity to administer power light at specific wavelengths to improve our body functions and counteract light deficiency (Dompe et al., 2020). One of these techniques is photobiomodulation (PBM) which uses red to infrared light, wavelengths between 600 and 1,100 nm, to stimulate, heal, regenerate and protect tissue (Hamblin, 2016; Mitrofanis and Jeffery, 2018). PBM is a non-invasive, inexpensive and safe technique (Gutiérrez-Menéndez et al., 2020).

At present, PBM therapy is widely used in several clinical conditions: diabetes, ulcers, blood disorders, coronary artery diseases, and musculoskeletal complications, among others, and presents well supported neurobiological effects, such as the improvement of wound healing, the regeneration of damaged tissues, and the reduction of pain and inflammation (Salehpour et al., 2019a; dos Santos Cardoso et al., 2021b). However, this therapy is not only used in peripheral tissues, it can also be applied to the nervous system. In this regard, the first evidence of PBM’s beneficial effects on the brain was after its application for ischemic stroke in different animal models (Hamblin, 2019). After that, numerous studies have documented a large number of positive brain effects resulting from this therapy, suggesting PBM as a new modality for neural activity stimulation to improve brain functions (Salehpour et al., 2018; Arias et al., 2019). The most supported outcomes of the use of PBM on the brain are the increase of intracellular ATP production, along with the improvement of metabolic function; changes in cerebral oxygenation and blood flow; anti-inflammatory effects; upregulation of anti-apoptotic proteins, increment of antioxidants and less excitotoxicity, the stimulation of neurons and glial cell neurogenesis, synaptogenesis, migration, and the secretion of brain neurotrophins (Hamblin, 2016, 2019; Dompe et al., 2020; Gutiérrez-Menéndez et al., 2020; dos Santos Cardoso et al., 2021a). The main proposed mechanism of action to achieve such changes is the stimulation of the cytochrome c oxidase (CCO), the terminal enzyme in the mitochondrial electron transport chain (Salehpour et al., 2019b; Dompe et al., 2020; Gutiérrez-Menéndez et al., 2021). However, PBM effects without adverse outcomes can only be reached with optimum parameters (wavelength, energy density, irradiance, area, mode of administration, etc.) and that is the current issue: the lack of consensus on which parameters are appropriate for each situation (Salehpour et al., 2018; Gutiérrez-Menéndez et al., 2020). Most of the studies support that the use of wavelengths between 810 nm and 1,064 nm in adult human and animal models of health and disease, are versatile and adequate to achieve several effects on brain activity and behavioral function (Gutiérrez-Menéndez et al., 2020).

The postnatal period is a critical phase for the development of the mammal brain. During this period, the dynamic gene expression and the methylation changes generate several effects on neurogenesis, synaptogenesis and neural circuit formation (Simmons et al., 2013). Studies using rat models have shown that the first three postnatal weeks are essential for the development of the neural system, with radical changes happening in terms of synaptic connectivity, neuroendocrine response and global neural gene expression (Simmons et al., 2013). Moreover, the cell energy metabolism is maturing and adult mitochondria characteristics are reached around postnatal day (PND) 30 (Kalous et al., 2001). Changes in BDNF mRNA and protein levels are also reported during this period, the profile of changes shows an increase in brain structures in the postnatal period and a decrease in the aging phase (Karege et al., 2002). All these processes allow immature brains which are particularly vulnerable to different insults (toxic, traumatic, vascular, etc.) to adapt (Calabrese et al., 2013).

Taking into account the high plasticity and the responsiveness of the postnatal brain, the use of PBM could be an effective technique for the modulation of the developing nervous system and could even be considered a potential treatment for several developmental disorders. Therefore, this study aims to analyze the effects of PBM on the young prefrontal cortex (PFC) and hippocampus of healthy male and female Wistar rats. Three groups between PND23 and PND29 of each sex were used: a PBM group that received 5 days of PBM, a device group that was submitted to the same conditions as the experimental group but without light radiation, and a control basal group. After PBM administration, we analyzed the three groups and compared each sex according to brain metabolic activity and the activation of immediate early genes through a CCO histochemistry and c-Fos immunostaining, respectively.

## Materials and Methods

### Animals

A total of 61 Wistar rats, 31 males and 30 females aged between 23 and 29 days old from the Oviedo University vivarium were used in this research. Animals were sorted by sex in transparent polycarbonate cages located in a room at a constant temperature (22 ± 2°C), 65–70% of relative humidity and an artificial light-dark cycle of 12 h (8:00–20:00/20:00–8:00). They had *ad libitum* access to food and tap water.

Animals were randomly separated by sex into three different groups: male (PBM; *n* = 10) and female (PBM; *n* = 10) PBM groups that received the radiation; male (PBMD; *n* = 10) and female (PBMD; *n* = 10) device PBM groups which were submitted to the same conditions as the PBM group but with the light switched off and finally, male (C; *n* = 11) and female (C; *n* = 10) control groups that were kept in their cages.

The animal manipulation and all the procedures carried out in this study were according to the European Communities Committee 2010/63/EU and Royal decree N° 53/2013 of the Ministry of the Presidency related to the protection of animals used for experimentation and other scientific purposes. The study was approved by the Ethics Committee of the Principality of Asturias.

### Photobiomodulation Therapy

The following procedure was carried out with both sexes for 7 days. The first 2 days, coinciding with PND22 and PND23, were devoted to getting the animal habituated to the researcher and the procedure. At PND22, animals were handled for 5 min and the first third of their head, just between the eyes, was shaved to maximize light penetration across prefrontal areas. The next day (PND23), animals were immobilized by the researcher one by one on a soft surface and the PBM device was placed on the shaved region for 10 min in OFF mode. This process was done 3 times until each animal had undergone 30 min. During the following 5 days (PND24-PND28), the same immobilized procedure was carried out, but the light device was in ON mode. DPBM and PBM groups were submitted to the same procedures, but the PBM device was kept OFF during all the studies in the DPBM group. A laser with a continuous wave at 810 nm wavelength was used for irradiation in PBM groups. The device was operated at an output power of 40 mW and irradiance of 65.6 W/m^2^, with a beam size of 0.0495 cm^2^. PBM groups received 36 cycles of PBM (40 s ON and 10 s OFF) reaching a total irradiation time of 24 min and an average fluence of 46.5 J/cm^2^ per day. Approximately 0.8% of the applied power reaches the brain tissue. This value was previously determined in rat skulls using a PM 160 optical power meter (ThorLabs, United States). Skull was placed on a bench designed for this purpose wherein at the top was the radiation laser and at the bottom, below the skull, the optical power meter.

### Tissue Processing

The day after finishing the light procedure, on PND29, animals were euthanized and brains were removed, frozen rapidly using *N*-methyl butane (Sigma-Aldrich, Madrid, Spain), and stored at −40^°^C. Coronal brain sections (30 μm) were cut at −20^°^C in a cryostat (Microm HM 505-E, Germany). Two series were obtained from each brain, one was mounted on non-gelatinized slides to conduct the CCO histochemistry and the other one on gelatinized slides to carry out the c-Fos immunostaining.

### Cytochrome C Oxidase Histochemistry

The procedure carried out for the tissue treatment was previously described by Zorzo et al. (2019). To quantify enzymatic activity and to control staining variability across the baths, sets of tissue homogenate standards from Wistar rat brains in PND29 were cut at different thicknesses (10, 30, 50, and 70 μm) and included with each bath of slides. Sections were fixed for 5 min using 0.1 M phosphate buffer (pH 7.6) with 10% (w/v) sucrose and 25% (v/v) glutaraldehyde. Then, three baths of 0.1 M phosphate buffer with 10% (w/v) sucrose were carried out for 5 min each, and one bath of 0.05 M Tris buffer, pH 7.6 for 8 min [0,275 mg/l cobalt chloride (Aldrich, Germany), 10% (w/v) sucrose (Sigma, Germany), 6 g/l Trizmabase (Sigma, United States), and 0.5 (v/v) dimethyl-sulfoxide (Sigma-Aldrich, Madrid, Spain)]. After that, sections and standards were maintained in a 0.1 M phosphate buffer, pH 7.6, for 5 min and incubated in a solution of 0.0075% (w/v) cytochrome c (Sigma-Aldrich, Madrid, Spain); 0.002% (w/v) catalase (Sigma, Spain); 5% (w/v) sucrose (Sigma, Germany); 0.25% (v/v) dimethyl-sulfoxide (Sigma-Aldrich, Madrid, Spain); and 0.05% (w/v) diaminobenzidine tetrahydrochloride (Sigma-Aldrich, Madrid, Spain) in 800 ml of 0.1 M phosphate buffer at 37°C for 1 h. Next, the reaction was stopped by fixing the tissue in a buffered 4% (v/v) formalin with a 0.1 M phosphate buffer, pH 7.6, 10% (w/v) sucrose, and 37% (v/v) formalin for 30 min at room temperature. Finally, the slides were dehydrated through a series of graded alcohols, cleared with xylene (Avanter, Poland), and cover slipped with Entellan (Merck, Germany).

### c-Fos Immunohistochemistry

Sections were fixed in a 0.1 M 4% (w/v) paraformaldehyde buffer (pH 7.4) for 30 min, in continuous agitation, and rinsed in two baths of 0.01 M phosphate-buffered saline (PBS) (pH 7.4). After that, they were incubated for 30 min with 3% (v/v) hydrogen peroxidase (Sigma-Aldrich, Madrid, Spain) in PBS (0.01 M, pH 7.4) to remove endogenous peroxidase activity, and washed twice in PBS (0.01 M, pH 7.4) for 10 min. Sections were maintained in a solution of 1% (v/v) Triton X-100 (Sigma-Aldrich, United States) in PBS (0.01 M, pH 7.4) for 10 min and, subsequently, washed in PBS (0.01 M, pH 7.4). After blocking with a phosphate buffer (0.01 M, pH 7.4) solution containing 3% (v/v) bovine serum albumin (Sigma-Aldrich, Madrid, Spain) for 30 min, sections were incubated with a rabbit polyclonal anti-c-Fos antibody solution (1:7,500) (Merck, Spain) diluted in a solution of PBS (0.01 M, pH 7.4), bovine serum albumin (Sigma-Aldrich, Madrid, Spain), and Triton X-100 (Sigma-Aldrich, United States) for 24 h at 4°C in a humid chamber. Next, slides were washed in two baths of PBS (0.01 M, pH 7.4) for 10 min each, and incubated in a goat anti-rabbit biotinylated IgG secondary antibody (Pierce, United States; diluted 1:480 in incubating solution) for 1 h at room temperature in a humid chamber. After two washes of PBS (0.01 M, pH 7.4) for 10 min each, sections were reacted with avidin-biotin-peroxidase complex (Vectastain ABC Ultrasensitive Elite Kit, Pierce, United States) for 1 h in a humid chamber. They were rinsed with two baths of PBS (0.01 M, pH 7.4), and the reaction was visualized by treating the sections for 4 min in a solution of PBS (0.01 M, pH 7.4), 0.05% w/v diaminobenzidine tetra-hydrochloride (Sigma-Aldrich, Madrid, Spain), 33% (v/v) hydrogen peroxidase solution and 0.05% (w/v) ammonium nickel (II) sulfate hexahydrate (Sigma-Aldrich, Madrid, Spain) in total darkness. The reaction was terminated by washing the sections twice in PBS (0.01 M, pH 7.4), and they were dehydrated through a series of graded alcohols, cleared with xylene (Avanter, Poland) and cover slipped with Entellan (Merck, Germany). All the immunohistochemistry procedures included sections that served as controls where the primary antibody was not added.

### Cytochrome C Oxidase Optical Density Quantification

The CCO histochemical intensity was quantified by densitometric analysis, using a computer-assisted image analysis workstation (MCID, Interfocus Imaging Ltd., Linton, England) which consisted of a high precision illuminator, a digital camera, and a computer with the specific image analysis software MDCID Core 7.0. The mean optical density (OD) of each region was measured using three consecutive sections in each subject. In each section, four non-overlapping readings were taken, using a square-shaped dissector adjusted for each region size. A researcher who was blind to the groups registered a total of 12 measurements per region/animal. Then, OD values were converted to CCO activity units, determined by the enzymatic activity of the standards measured spectrophotometrically.

We defined the regions of interest according to the atlas of Paxinos and Watson (2007). The regions and their distances in mm counted from bregma were: +3.24 mm for the cingulate cortex (CG), prelimbic cortex (PL) and infralimbic cortex (IL) and -3.24 mm for the CA1, CA3, and the dentate gyrus (DG) subfields of the dorsal hippocampus.

### c-Fos Cells Counting

Quantification was performed by systematically sampling each selected region using counting frames superimposed over the region with a microscope (Leica Microsystems DFC490, Germany) coupled to a computer with the Leica Application Suite X software (Leica Microsystems, Germany) with a total magnification of 192X. The sizes of the counting frames were 250,000 μm for CG, PL and IL and 72,000 μm for CA1, CA3 and DG. The total area sampled by these frames per region in each section was: 500,000 μm for CG, PL and IL; 144,000 μm for CA1 and CA3 and finally, 72,000 μm for DG. c-Fos-positive nuclei were defined based on homogenous gray-black stained elements with a well-defined border. Finally, the mean c-Fos positive nuclei count in two sections was calculated for each subject and region.

The regions of interest and their distances (mm) counted from bregma according to the atlas of Paxinos and Watson (2007) were: +3.24 mm for the cingulate cortex (CG), prelimbic cortex (PL) and infralimbic cortex (IL) and -3.24 mm for the CA1, CA3, and the dentate gyrus (DG) sub-fields of the dorsal hippocampus. In these regions, we quantified the number of c-Fos positive nuclei in two alternate sections 30 μm apart. The slides were coded so that the researcher who performed the entire analysis did not know the treatment of the individual subjects.

### Statistical Analysis

The data were analyzed using the SigmaPlot 12.5 program (Systat, Richmond, United States). Differences were considered statistically significant when *p* < 0.05. We used a Shapiro-Wilk test to test the normality assumption (*p* > 0.05). When the data fit a normal distribution, we used parametric tests. Otherwise, we used non-parametric tests. The SigmaPlot 12.5 software program (Systat, Richmond, CA, United States) was also used for the graphic representation of the results. We presented data as mean + SEM.

### Cytochrome C Oxidase Results

Statistical groups comparisons (PBM, PBMD and C) of CCO activity in each sex were analyzed using a one-way ANOVA for each region of interest. We carried out a two-way ANOVA *[Sex × Group (PBM, PBMD and C)]* to examine differences between sexes for each region of interest. *Post hoc* comparisons using the Holm-Sidak method were carried out when significant differences were found.

### c-Fos Activity

The results of the c-Fos quantification were expressed as the average of c-Fos positive cells/μm^2^ for the two consecutive sections of each region of interest. We analyzed differences between the three groups (PBM, DPBM and C) in each sex using a one-way ANOVA. Additionally, a two-way ANOVA *[Sex x Group (PBM, PBMD and C)]* was performed to examine c-Fos activity differences between sexes for each region of interest. Holm-Sidak method was used when significant differences were found.

## Results

### Cytochrome C Oxidase Activity

**FIGURE 1:**
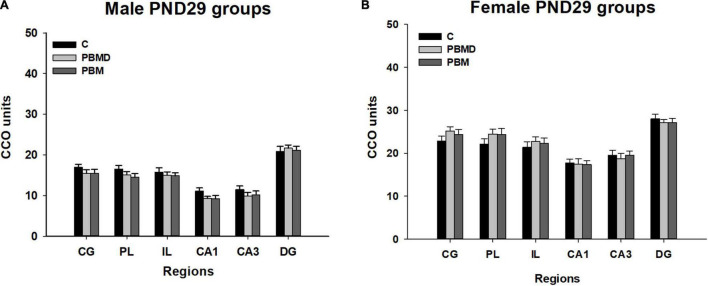
CCO results (mean ± SEM). **(A)** CCO values in 29-day-old male groups (PBM, PBMD, C). There were no differences between the groups in the assessed areas (*p* > 0.05). **(B)** CCO values in 29 day-old female groups (PBM, PBMD, C). No significant differences were found in any of the areas (*p* > 0.05). Groups: C, control group; PBMD, photobiomodulation device group; PBM, photobiomodulation group. Areas: CG, Cingulate cortex; PL, Prelimbic cortex; IL, Infralimbic cortex; CA1, field CA1 of hippocampus; CA3, field CA3 of hippocampus; DG, Dentate Gyrus. PND, postnatal day; CCO, cytochrome c oxidase.

**FIGURE 2:**
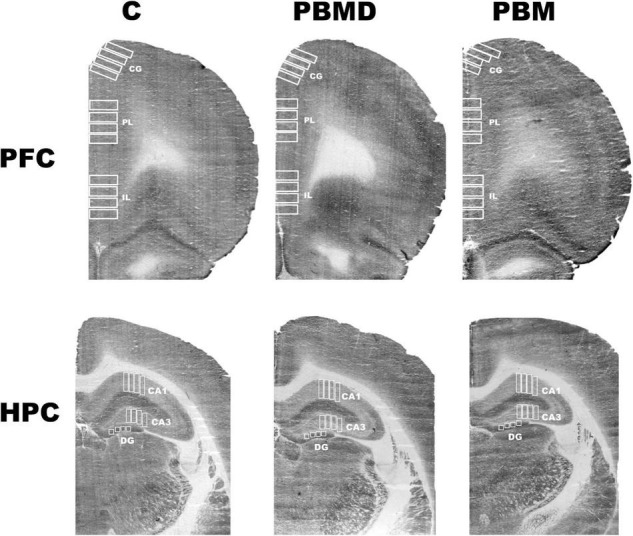
CCO samples. Representative photograph of the CCO optical density of the three groups (C, PBMD, and PBM) in PFC and HPC. There were no differences between groups in any of the studied regions in male or female samples. Groups: C, control group; PBMD, photobiomodulation device group; PBM, photobiomodulation group. Areas: CG, Cingulate cortex; PL, Prelimbic cortex; IL, Infralimbic cortex; CA1, field CA1 of hippocampus; CA3, field CA3 of hippocampus; DG, Dentate Gyrus. PFC, prefrontal cortex; HPC, hippocampus; PND, postnatal day; CCO, cytochrome c oxidase.

**FIGURE 3:**
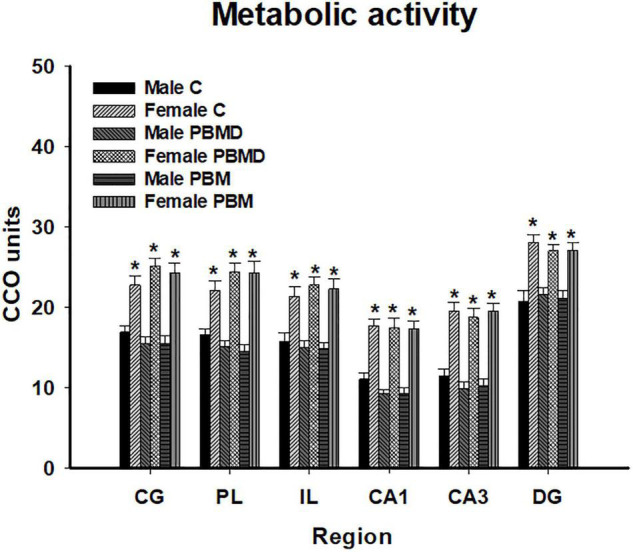
CCO differences between sexes in the three groups (PBM, PBMD, and C) in each region of interest (mean ± SEM). Female groups showed a general pattern of higher metabolic activity in all the studied areas (*p < 0.05). Groups: C, control group; PBMD, photobiomodulation device group; PBM, photobiomodulation group. Areas: CG, Cingulate cortex; PL, Prelimbic cortex; IL, Infralimbic cortex; CA1,field CA1 of hippocampus; CA3, field CA3 of hippocampus; DG, Dentate Gyrus. PFC, prefrontal cortex; HPC, hippocampus; PND, postnatal day; CCO, cytochrome c oxidase.

The analysis of the metabolic activity in male and female groups showed the same pattern of CCO activity in both sexes. The three studied groups (PBM, PBMD, and C) did not show CCO differences in any of the regions of interest in the male group [CG: *F*_(2, 27)_ = 0.973, *p* = 0.391; PL: *F*_(2, 28)_ = 1.677, *p* = 0.205; IL: *F*_(2, 28)_ = 0.283, *p* = 0.756; CA1: *F*_(2, 27)_ = 2.268, *p* = 0.123; CA3: *F*_(2, 27)_ = 0.901; *p* = 0.418; DG: *F*_(2, 27)_ = 0.162, *p* = 0.851; Figure 1A] or in the female group [CG: *F*_(2, 28)_ = 1.112, *p* = 0.343; PL: *F*_(2, 28)_ = 1.196, *p* = 0.317; IL: *F*_(2, 28)_ = 0.366, *p* = 0.697; CA1: H_2_ = 0.294, *p* = 0.864; CA3: *F*_(2, 28)_ = 0.161; *p* = 0.852; DG: *F*_(2, 28)_ = 0.322, *p* = 0.727; Figures 1B, 2]. Regarding the analysis of the differences in CCO activity between sexes, the two-way ANOVA showed that only the *Sex* factor was significant in all the studied regions [CG: *F*_(1, 55)_ = 96.286, *p* < 0.001; PL: *F*_(1, 56)_ = 92.622, *p* < 0.001; IL: *F*_(1, 56)_ = 64.901, *p* < 0.001; CA1: *F*_(1, 56)_ = 123.085, *p* < 0.001; CA3: *F*_(1, 56)_ = 115.807, *p* < 0.001; DG: *F*_(1, 56)_ = 58.533, *p* < 0.001]. *Post hoc* comparisons showed a general pattern of higher metabolic activity in the female groups in all the studied regions [CG: *t* = 9.813, *p* < 0.001; PL: *t* = 9.624, *p* < 0.001; IL: *t* = 8.056, *p* < 0.001; CA1: *t* = 11.094, *p* < 0.001; CA3: *t* = 10.761, *p* < 0.001; DG: *t* = 7.651, *p* < 0.001; Figure 3].

### c-Fos Results

**FIGURE 4:**
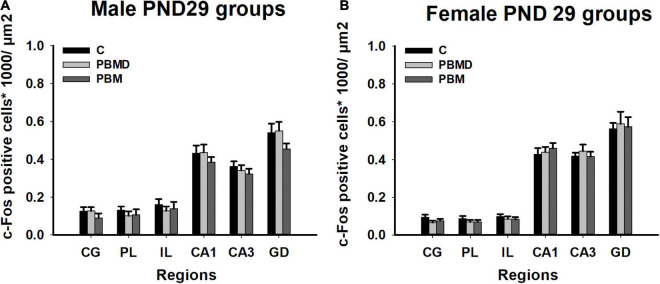
c-Fos-positive cells (positive cells of c-Fos/μm^2^) results in the regions of interest. **(A)** c-Fos results in 29 day-old male groups (C, PBMD, and PBM). No significant differences were found in any of the areas (*p* > 0.05). **(B)** c-Fos results in 29 day-old female groups (C, PBMD, PBM). There were no differences between the groups in the assessed areas (*p* > 0.05). Groups: C, control group; PBMD, photobiomodulation device group; PBM, photobiomodulation group. Areas: CG, Cingulate cortex; PL, Prelimbic cortex; IL, Infralimbic cortex; CA1, field CA1 of hippocampus; CA3, field CA3 of hippocampus; DG, Dentate Gyrus. PND, postnatal day.

**FIGURE 5:**
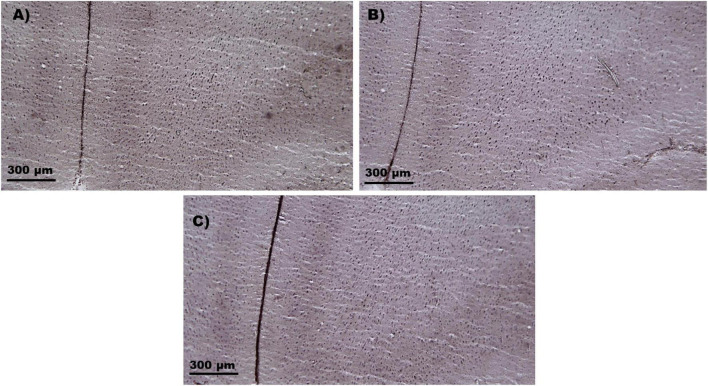
c-Fos samples. Representative microphotograph of the c-Fos immunostaining in the IL cortex. **(A)** c-Fos sample of the control group. **(B)** c-Fos sample of the photobiomodulation device group. **(C)** c-Fos sample of the photobiomodulation group. No differences in c-Fos protein expression were found between the three groups in the male or female groups. IL, Infralimbic cortex.

**FIGURE 6:**
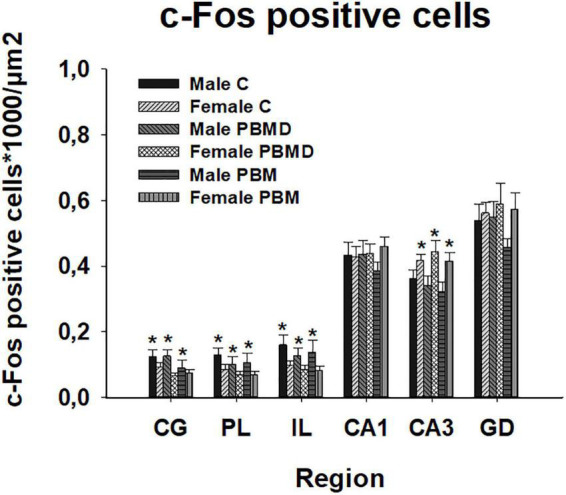
c-Fos positive cells (positive cells of c-Fos/μm^2^) differences between sexes in the three groups (PBM, PBMD, and C) in each region of interest (mean ± SEM). Male groups showed more c-Fos positive cells in the prefrontal cortex (CG, IL, and PL) (*p < 0.05) while female groups showed higher c-Fos expression in CA3 (*p < 0.05). Groups: C, control group; PBMD, photobiomodulation device group; PBM, photobiomodulation group. Areas: CG, Cingulate cortex; PL, Prelimbic cortex; IL, Infralimbic cortex; CA1, field CA1 of hippocampus; CA3, field CA3 of hippocampus; DG, Dentate Gyrus.

The number of c-Fos positive nuclei, as in the CCO analysis, reported no differences between any of the three groups (PBM, PBMD, and C) in the male [CG: *F*_(2, 19)_ = 0.869, *p* = 0.425; PL: *F*_(2, 19)_ = 0.451, *p* = 0.644; IL: *F*_(2, 19)_ = 0.304, *p* = 0.741; CA1: *F*_(2, 27)_ = 0.571, *p* = 0.572; CA3: *F*_(2, 27)_ = 0.522; *p* = 0.599; DG: *F*_(2, 27)_ = 1.500, *p* = 0.241; Figure 4A] or female groups [CG: H_2_ = 3.144, *p* = 0.208; PL: *F*_(2, 26)_ = 0.680, *p* = 0.516; IL: *F*_(2, 26)_ = 0.373, *p* = 0.692; CA1: *F*_(2, 26)_ = 0.279, *p* = 0.759; CA3: *F*_(2, 26)_ = 0.363; *p* = 0.699; DG: *F*_(2, 26)_ = 0.069, *p* = 0.933; Figure 4B] in any of the quantified regions (Figure 5). Regarding the differences in c-Fos positive nuclei between sexes, results showed that only the *Sex* factor was significant in CG [*F*_(1, 45)_ = 7.544, *p* = 0.009], PL [*F*_(1, 45)_ = 6.939, *p* = 0.012], IL [*F*_(1, 45)_ = 9.186, *p* = 0.004], and CA3 [*F*_(1, 53)_ = 13.919, *p* < 0.001] but not in CA1 [*F*_(1, 53)_ = 0.738, *p* = 0.394] and DG [*F*_(1, 53)_ = 2.326, *p* = 0.133]. *Post hoc* comparisons showed more c-Fos positive nuclei in male groups in CG (*t* = 2.747, *p* = 0.009), PL (*t* = 2.634, *p* = 0.012) and IL (*t* = 3.031, *p* = 0.004) while, in contrast, female groups had greater c-Fos expression in CA3 (*t* = 3.731, *p* < 0.001) (Figure 6).

## Discussion

To our knowledge, this is the first research to examine PBM effects in the postnatal brain of healthy male and female rats. In our study, we applied an 810 nm near-infrared light to the scalp surface over the prefrontal region of 24 day-old male and female Wistar rats in order to analyze its potential alterations in brain metabolic activity and c-Fos protein expression. Results showed that both brain activity markers were not disturbed after the use of light in any of the sexes.

PBM is a relatively new technique that uses light in the red or red infrared range to heal, restore, stimulate physiological processes, normalize cellular function, and repair injury or disease damages (de Pauli Paglioni et al., 2019; Ramezani et al., 2021). The most support mechanism of action is the light particles absorption by the CCO enzyme particularly in stressed/damaged cells (Martin et al., 2021). CCO is localized in the electron transport chain of the mitochondrial membrane and it is involved in the production of the main energy molecule, the adenosine triphosphate (ATP) (Gutiérrez-Menéndez et al., 2020). PBM has been employed since the 1990s for therapeutic purposes such as inflammatory, infectious, traumatic or autoimmune lesions (de Pauli Paglioni et al., 2019). At present, PBM is also being used in the treatment of many pathological disorders and diseases such as Parkinson’s and Alzheimer’s diseases, depression, traumatic brain injury, etc., as a non-invasive non-thermal and painless therapy, achieving positive cognitive and brain effects (Meynaghizadeh-Zargar et al., 2019; Salehpour and Hamblin, 2020; Salehpour et al., 2021). Despite light absorption is has supposed to occur particularly in stressed/damaged cells, it has been also studied the effects of this technique on healthy adult subjects with no clinical symptoms, finding positive effects. Human research reported an overall improvement in cognitive functions after the use of PBM most of them by using a wavelength of 1,064 nm (Barrett and Gonzalez-Lima, 2013; Blanco et al., 2016, 2017; Hwang et al., 2016; Gonzalez-Lima, 2017; Wang et al., 2017; Gonzalez-Lima et al., 2019; Holmes et al., 2019; Saucedo et al., 2021). Regarding brain activity markers, Gonzalez-Lima (2017) found an increase of CCO and oxygenated hemoglobin concentrations in the prefrontal cortex and Wang et al. (2017) and Zomorrodi et al. (2019) showed variations in the brain waves frequency. Moreover, healthy animal studies achieved better behavioral outcomes and brain changes after PBM radiation (Michalikova et al., 2008; Arias et al., 2020; Gutiérrez-Menéndez et al., 2021; Table 1).

**TABLE 1:** Comparison of PBM parameters used in previous research.

References	Sample	Age period	PBM device	Wave type	Wavelength (nm)	Irradiance (mW/cm^2^)
Arias et al. (2016)	Portal hypertension animals	Adulthood	LED	–	610 ± 10	50
Arias et al. (2020)	Healthy animals	Adulthood	LED	–	610 ± 10	50
Banqueri et al. (2019)	Early stressed animals	Adulthood	Laser	–	1,064	(Power: 30 mW)
Barrett and Gonzalez-Lima (2013)	Healthy humans	Adulthood	Laser	Continuous	1,064	250
Blanco et al. (2016, 2017)	Healthy humans	Adulthood	Laser	Continuous	1,064	250
Buzzá et al. (2019)	Healthy animal	Newborn	LED	–	630	4
De Taboada and Hamblin (2019)	Alzheimer’s disease animal model	Adulthood	Laser	Pulsed	810	(Powers: 40, 200, and 400 mW)
dos Santos Cardoso et al. (2021a)	Healthy animals	Adolescence/Adulthood	Laser	Continuous	810	(Power: 100 mW)
Gonzalez-Lima (2017); Gonzalez-Lima et al. (2019)	Healthy humans	Adulthood	Laser	Continuous	1,064	250
Gutiérrez-Menéndez et al. (2021)	Healthy animals	Adulthood	Laser	–	1,064	(Power: 30 mW)
Holmes et al. (2019)	Healthy humans	Adulthood	Laser	–	1,064	250
Hwang et al. (2016)	Healthy humans	Adulthood	Laser	–	1,064	250
Li et al. (2021)	Posttraumatic stress disorder animal model	Adulthood	Laser	Continuous	808	25
Méndez et al. (2021)	Hepatic encephalopathic animals	Adulthood	LED	–	610 ± 10	50
Michalikova et al. (2008)	Healthy animals	Adulthood	Laser	Continuous	1,072	–
Nadur-Andrade et al. (2016)	Bothrops moojeni venom in animals	Adulthood	Laser	–	685	(Power: 30 mW)
Saucedo et al. (2021)	Healthy humans	Adolescence/Adulthood	Laser	Continuous	1,064	250
Shinhmar et al. (2020)	Healthy humans	Adulthood	LED	–	670	40
Shinhmar et al. (2020)	Healthy humans	Adulthood	Laser	–	1,064	160
Zomorrodi et al. (2019)	Healthy humans	Adulthood	LED	Pulsed	810	75, 25, and 100

The development of the nervous system depends on the continuous interaction of several processes that start during the fetal period. Some of these developmental processes are completed before birth and others continue in the postnatal period, extending into adulthood (Tsujimoto, 2008). The postnatal stage is a sensitive period of brain development. It is characterized by the presence of neurogenesis and gliogenesis, cell’s migration and differentiation, and the rapid formation of synapses (synaptogenesis) and their remodeling and elimination (Bandeira et al., 2009; Miki et al., 2014; Chen et al., 2017). However, all these processes do not start at the same time. Individual brain regions have been shown to have individual time scales for maturation, being the PFC and also the hippocampus ones of the latest (Tsujimoto, 2008; Mengler et al., 2014). Likewise, cell metabolism also matures after birth. Mitochondria exhibit an increment of CCO activity and higher content of cytochrome in the first month of postnatal life, increasing their oxidative capacity (Kalous et al., 2001). According to these claims and taking into account all the positive PBM outcomes on brain function and activity, the potential use of this technique not only in healthy adult brains but also in developing stages is not surprising. However, little research is focused on PBM administration in these periods. In order to contribute to increasing the knowledge of the effects of PBM in the postnatal period, we applied 5 days of PBM therapy in healthy male and female Wistar rats starting at PND 24. As we mentioned before, the choice of the light parameters is an essential component that we have to consider in the use of this technique. In our study, we choose a near-infrared light of 810 nm due to the high support of this wavelength from other research (Gutiérrez-Menéndez et al., 2020; dos Santos Cardoso et al., 2021a), as well as previous pilot studies in young subjects that our team carried out. Regarding the treatment duration and the way of application, we decided to administer PBM manually for 30 min in order to reduce stress during the application. After PBM administration, we analyzed differences between groups according to metabolic activity and the activation of immediate early genes in each sex. In this developmental stage, there is an increment of cytochrome content and CCO activity (Kalous et al., 2001). As PBM has its potential effect on the CCO, as a result, its redox status and its functional activity would be modulated (Karu, 2014; Dompe et al., 2020) so in our research, changes in the metabolic activity of our subjects would be expected. However, we did not find CCO differences between male groups nor female groups in the PFC or the hippocampus. Little research has focused specifically on the analysis of the metabolic activity after PBM administration. Most of the studies were carried out using adult samples and they support the idea that the photon absorption by CCO leads to an increase in enzyme activity, oxygen consumption and ATP production due to the photodissociation of inhibitory nitric oxide (Gonzalez-Lima, 2017; Hamblin, 2017, 2019; Hennessy and Hamblin, 2017; Arias et al., 2020). However, the contrary effect was also found, a reduction in the oxidative metabolic activity in several limbic regions of adult control rats after PBM administration (Gutiérrez-Menéndez et al., 2021; Table 1). It should be noted that despite the results being directly dependent on which parameters have been chosen and there was high variability between these studies (Gutiérrez-Menéndez et al., 2020), PBM administration achieves CCO modifications in adult control subjects. Moreover, CCO changes after PBM have been identified in adult healthy subjects, in several models of disease and also in cognitive tasks, reporting a general decrease in metabolic activity in the radiated groups (Arias et al., 2016; Banqueri et al., 2019; Gutiérrez-Menéndez et al., 2021; Méndez et al., 2021). Nevertheless, in our study, after the application of PBM in young healthy subjects, we did not find any alterations in metabolic activity in male or female groups Additionally, the analysis of the metabolic activity differences between sexes in the three groups (PBM, PBMD, C), showed higher CCO activity in the three female groups than male groups in the prefrontal cortex and the hippocampus. These results are according with the previous study of Spivey et al. (2008) that showed lower male regional metabolic activity in prefrontal and parietal cortex of healthy juvenile rats compared to the juvenile female group. Similar results using adult samples were found by González-Pardo et al. (2020). In the same way, the c-fos proto-oncogene expression has been less studied after the application of PBM. This immediate early gene is one of the first groups of genes that express within minutes after synaptic and neuronal activation triggered by extracellular stimulation and it is involved in cell proliferation and differentiation (Velazquez et al., 2015; Li et al., 2021). As in the CCO studies, results are controversial: several research studies have achieved an increase of c-Fos protein expression but others found a decrease of its expression in several brain areas in healthy and disease models (Nadur-Andrade et al., 2016; De Taboada and Hamblin, 2019; Arias et al., 2020; Li et al., 2021; Table 1). By contrast, in our study, we did not achieve any alterations in the c-Fos protein expression between young groups in males or females after light application. However, differences in the c-Fos expression between sexes in the three groups (PBM, PBMD, and C) were found. The three male groups displayed greater c-Fos positive cells than females in the three studied areas of the prefrontal cortex while, in contrast, female groups showed higher c-Fos expression in the CA3 subregion of the hippocampus.

Our results are in accordance with the studies of Shinhmar et al. (2020), dos Santos Cardoso et al. (2021a), and Saucedo et al. (2021) (Table 1), who compared light administration effects on retinal function, CCO and hemodynamic activity and neuroinflammatory response, respectively, between young and aged subjects. They found that after radiation, PBM effects were greater in the older subject groups. It is known that in brain aging several neurodegeneration processes, such as local inflammation and energy metabolism reduction, take place (dos Santos Cardoso et al., 2021b,c). Therefore, these researchers speculated that the age-related mitochondrial decline, which is not present in young populations, plays a key role in the outcomes of PBM therapy (Shinhmar et al., 2020; dos Santos Cardoso et al., 2021a; Saucedo et al., 2021). The same reasons can explain our findings, as we applied PBM therapy in young subjects without any apparent health issues and in the developing brain. At this stage, brain function, specifically brain mitochondrial function, is not disturbed so, the action of PBM in the mitochondria is not detectable using the analysis of CCO activity and c-Fos protein expression. We can suspect that PBM therapy would be more effective in mitochondrially-compromised individuals such as in adult/older subjects and even in several diseases, than in young healthy subjects (Scaglia, 2010; Saucedo et al., 2021). Nevertheless, there is a study by Buzzá et al. (2019) which found high weight, faster eye-opening, and normal blood count in the developmental postnatal stage after applying red light in postnatal rats from PND2 until PND13. Despite the chosen subjects being healthy and newborn rats, results showed earlier maturity without damage in the radiated group, showing the potential applications of PBM in the first developing stages (Buzzá et al., 2019; Table 1). The study of Buzzá et al. (2019) used PBM at an earlier developmental stage compared to the present study and did not examine any brain modification, differing from our functional assessment of metabolic activity’s and c-Fos expression’s alterations. However, it should be pointed out that our study has several limitations that could lead to the results we found. The analysis of CCO and c-Fos variations could be not enough to detect PBM changes in these healthy young brains and the addition of other functional or behavioral methods could have helped to show PBM effects. Additionally, despite we included a device group submitted to the same conditions as the PBM group but without the light radiation for each sex in an attempt to control any stress influences, the inclusion of a positive control group would be relevant to show that the histological methods used are sensitive to changes. These markers of brain function suffer modifications in demanding conditions such as in an early stress period (Banqueri et al., 2019) or under cognitive requirements (Gonzalez-Lima, 2017; Gutiérrez-Menéndez et al., 2021; Table 1).

In conclusion, we applied 5 days of 810 nm-PBM therapy in the frontal area of the brain of male and female rats from PND 24 to PND 28 and we did not find any changes in metabolic activity nor c-Fos protein expression in any of the studied groups in the PFC nor the hippocampus. Taking into account the positive effects reported in the developmental stage in healthy subjects, the analysis of CCO and c-Fos variations carried out in our study could be not enough to detect PBM changes in these healthy young brains. More studies are necessary to examine in depth PBM outcomes in brain development, cognitive functions and postnatal disorders, along with the exploration of the optimal light parameters.

## Data Availability Statement

The original contributions presented in the study are included in the article/supplementary material, further inquiries can be directed to the corresponding author/s.

## Ethics Statement

The animal study was reviewed and approved by the Ethics Committee of the Principality of Asturias.

## Author Contributions

AG-M, MM, and JA designed the experiments. JM designed and developed the PBM device. AG-M performed the experiments, analyzed the data, and drafted the manuscript. MM and JA helped with the experimental steps. All authors revised the manuscript and approved its final version to be published and agreed to be accountable for all aspects of the manuscript.

## Conflict of Interest

The authors declare that the research was conducted in the absence of any commercial or financial relationships that could be construed as a potential conflict of interest.

## Publisher’s Note

All claims expressed in this article are solely those of the authors and do not necessarily represent those of their affiliated organizations, or those of the publisher, the editors and the reviewers. Any product that may be evaluated in this article, or claim that may be made by its manufacturer, is not guaranteed or endorsed by the publisher.
